# Repulsive response of *Meloidogyne incognita* induced by biocontrol bacteria and its effect on interspecific interactions

**DOI:** 10.3389/fmicb.2022.994941

**Published:** 2022-09-15

**Authors:** Yanli Zhao, Qinying Zhou, Chenggang Zou, Keqin Zhang, Xiaowei Huang

**Affiliations:** ^1^State Key Laboratory for Conservation and Utilization of Bio-Resources, and College of Life Science, Yunnan University, Kunming, China; ^2^Institute of Medicinal Plants, Yunnan Academy of Agricultural Sciences, Kunming, China; ^3^School of Medicine, Yunnan University, Kunming, China

**Keywords:** plant parasitic nematodes, *Meloidogyne incognita*, biocontrol bacteria, repulsive response, host plant, interspecific interactions

## Abstract

The aversive behavior of *Caenorhabditis elegans* is an important strategy that increases their survival under pathogen infection, and the molecular mechanisms underlying this behavior have been described. However, whether this defensive response occurs in plant-parasitic nematodes (PPNs), which have quite different life cycles and genomic sequences from the model nematode, against biocontrol microbes and affects interspecific interactions in ecological environments remains unclear. Here, we showed that *Meloidogyne incognita*, one of the most common PPNs, engaged in lawn-leaving behavior in response to biocontrol bacteria such as *Bacillus nematocida* B16 and *B. thuringiensis* Bt79. Genomic analysis revealed that the key genes responsible for the aversive behavior of *C. elegans*, such as serotonin-and TGF-β-related genes in canonical signaling pathways, were homologous to those of *M. incognita*, and the similarity between these sequences ranged from 30% to 67%. Knockdown of the homologous genes impaired avoidance of *M. incognita* to varying degrees. Calcium ion imaging showed that the repulsive response requires the involvement of the multiple amphid neurons of *M. incognita*. *In situ* hybridization specifically localized *Mi-tph-1* of the serotonin pathway to ADF/NSM neurons and *Mi-dbl-1* of the TGF-β pathway to AVA neurons. Our data suggested that the repulsive response induced by different biocontrol bacteria strongly suppresses the invasion of tomato host plants by *M. incognita*. Overall, our study is the first to clarify the pathogen-induced repulsive response of *M. incognita* and elucidate its underlying molecular mechanisms. Our findings provide new insights into interspecific interactions among biocontrol bacteria, PPNs, and host plants.

## Introduction

Plant parasitic nematodes (PPNs) are some of the most devastating agricultural pests. Root-knot nematodes (*Meloidogyne* spp.) are the most omnivorous PPNs, and they can parasitize more than 5,500 host plants and cause over $100 billion agriculture losses worldwide annually (Wang et al., 2021b). *Meloidogyne* preferentially invades plant roots and forms root galls, which impede the normal uptake of water and nutrients, and further facilitates the infection of some other soil-borne phytopathogens (Makhubu et al., 2021).

PPNs accomplish their life cycles through a robust chemosensory system, which allows them to sense a variety of chemical signals from soil and plants, and their responses to these signals are mediated *via* the synergistic functions of chemoreceptors, neuropeptides, and molecular signaling pathways. For example, root exudates secreted by plants strongly attract the second-stage juveniles (J2s) of *Meloidogyne*, which play an indispensable role when nematodes migrate in soil and search for suitable feeding sites on plants (Oota et al., 2020). During this process, chemical gradients of volatile chemicals such as pinene, limonene, and tridecane, and organic acids, such as malic acid, oxalic acid, and lactic acid have been reported to be tracked by *Meloidogyne* (Wang et al., 2021a). *Meloidogyne* also shows a chemotactic response toward fats and their derivatives, including low concentrations of lauric acid from crown daisy (Dong et al., 2014). A few phytohormones, such as zeatin, ethylene, jasmonic acid, salicylic acid, and abscisic acid, contribute to the development of feeding cells and alter the attractiveness of plant roots (Wubben et al., 2004; Swiecicka et al., 2009; Fudali et al., 2013; Cabrera et al., 2014; Kirwa et al., 2018). However, repellents, such as dibutyl phthalate, palmitic acid and linoleic acid derived from plant roots, induce repulsive response in *M. incognita* J2 and thus provide protection against parasitism by PPNs (Yang et al., 2016; Dong et al., 2018).

In *C. elegans*, pathogen-induced aversive behavior is an important defense strategy that confers resistance to virulent bacteria, in addition to innate immunity. Pathogen-induced aversive behavior also keeps the nematodes away from predators or tainted food. Several canonical molecular signaling pathways, such as the serotonin or TGF-β pathways, are responsible for the pathogen-induced aversive behavior of *C. elegans* (Shivers et al., 2009; Melo and Ruvkun, 2012; Zhang and Zhang, 2012). However, whether PPNs use their chemosensory system to induce the similar repulsive response in the presence of biocontrol bacteria, or whether this response affects their ability to infect host plants and subsequently alter the efficiency of biocontrol agents remains unclear (Zhou et al., 2019).

In 2008, the draft genomes of *M. incognita* and *M. hapla* were published, and bioinformatic analyses have suggested that the size of the genomes of these two root-knot nematodes is significantly reduced compared with the genome of *C. elegans*; this reduction is especially pronounced for families of genes involved in stress responses and innate immunity, such as glutathione transferase, cytochrome P450, immune effector elements, G protein-coupled receptors, and chitin-degrading enzymes, which might be attributed to the parasitic lifestyle of *Meloidogyne* within host plants (Abad et al., 2008; Opperman et al., 2008). The significant differences in the genome sequences of *Meloidogyne* and *C. elegans* suggest that the repulsive response of *M. incognita* might differ from that of *C. elegans*, or even be stronger than that of *C. elegans* to compensate for the loss of genes involved in stress resistance and innate immunity. Previous studies had shown that *Bacillus* and *Pseudomonas* spp. were the most common populations that colonized the rhizosphere and could effectively antagonize root-knot nematodes for their nematocidal activity (Zhu et al., 2021; Antil et al., 2022; Gowda et al., 2022). Here, the repulsive response of *M. incognita* against two species of biocontrol bacteria, including *B. nematocida* B16 and *B. thuringiensis* Bt79, was investigated and the signal pathways underlying this behavior were elucidated. Our findings provide new insights into interspecific interactions among biocontrol bacteria, PPNs, and host plants and have implications for the development of new biocontrol strategies.

## Materials and methods

### Preparation of *Meloidogyne incognita*

A pure isolate of *M. incognita* was maintained on tomatoes (*Solanum lycopersicum* cv. Jingfen Champion) in the greenhouse at Yunnan University, Kunming, China. Egg masses were collected from infected plants and hatched in distilled water. The freshly hatched *M. incognita* J2s were used in subsequent experiments.

### Repulsive response assay

**Figure 1:**
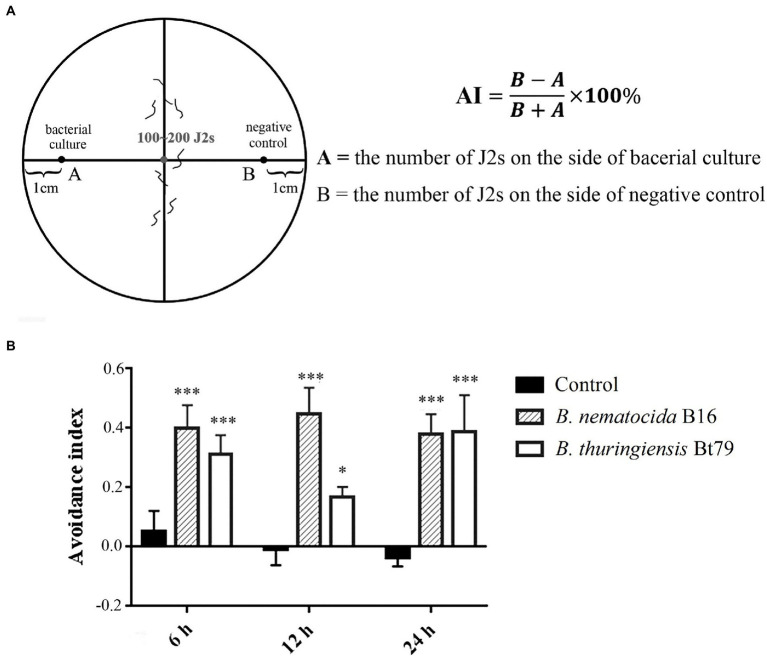
Repulsive response of *Meloidogyne incognita* second-stage juveniles (J2s) induced by different biocontrol bacteria. **(A)** Schematic diagram of the chemotaxis assay of *M. incognita* J2s on PF-127 medium. **(B)** Avoidance index of *M. incognita* J2s against different biocontrol bacteria. ^*^p < 0.05 and ^***^p < 0.001.

Chemotactic assays were used to test the repulsive response of *M. incognita* on pluronic gel medium of PF-127 (Sigma-Aldrich, St. Louis, United States; Figure 1A). In the avoidance assay, 4 ml of 23% (w/v) PF-127 (liquid at 4°C) was added to a 6 cm diameter Petri dish at room temperature for the gel to solidify. Five μl of bacterial culture and LB fluid medium without bacteria, which were used as the sample and control groups, respectively, were added to opposite sides at a distance of 2 cm from the center of the plate. Approximately 100 *M. incognita* J2s were added to the center of the plate, and the lid was closed. After 6, 12, and 24 h, the numbers on both sides were counted under stereomicroscope (Nikon Co, Tokyo, Japan). The avoidance index (AI) was calculated using the following formula: (number of nematodes on the control side – number of nematodes on the sample side) /total number of nematodes; the index ranged from −1.0 to 1.0 (Shivakumara et al., 2018; Kuang et al., 2020). Locomotion, including the crawling pattern and movement tracks, was also assessed on PF-127 medium under the stereomicroscope.

### Calcium imaging

Fura-2-AM (Beyotime, Shanghai, China), a fluorescent indicator with high affinity for Ca^2+^, was used to detect the intracellular Ca^2+^ concentration (Shuang et al., 2010). Approximately 300 freshly hatched J2s were soaked in 500 μl of M9 buffer containing 2 μl of 20 mM Fura-2-AM and 10 μl of 0.5% resorcinol, and they were incubated in the dark at room temperature on a rotary mixer for 6 h and then washed several times with sterile M9 buffer. A fluorescent microscope of Nikon E800 (Nikon Co, Tokyo, Japan) was used to record the fluorescence intensity in the head neurons under 330–350 nm excitation wavelengths before and after the bacterial culture of *B. nematocida* B16 and *B. thuringiensis* Bt79 was added near J2. The software ImageJ was used to quantify the mean fluorescence intensity.

### cDNA cloning of target genes from *Meloidogyne incognita*

Total RNA was extracted from 10,000 J2s by Trizol reagent (Tiangen Co., Tianjin, China). Synthesis of cDNA was performed using random primers with the Prime Script RT Reagent Kit (Takara, Dalian, China). The target cDNA of *Mi-tph-1*, *Mi-mod-1*, *Mi-dbl-1*, and *Mi-sma-6* was amplified using EX Taq DNA polymerase (Takara, Dalian, China). The components of the PCR reaction were 0.5 μl of each primer, 0.5 μl of cDNA, 2.5 μl of 10 × Ex Taq buffer, 2 μl of dNTP mix, 0.2 μl of Ex Taq DNA Polymerase, and 18.8 μl of double distilled water. The primers are shown in Table 1. The amplified fragment was then cloned into pGEM-T Easy Vector (Promega, Madison, United States), and the inserted fragment was confirmed by sequencing.

**Table 1:** The primers used to amplify cDNA of the target genes or the dsDNA probes for ISH.

Gene	Primer sequence 5′–3′
*Mi-tph-1*	GCAGAACTGTTGCTCCTCAT
*Mi-tph-1* R	AGCTACTGGACGTACACGAA
*Mi-mod-1*	GCAAGACATGGGCTCGTTAA
*Mi-mod-1* R	GGGACATAAGCCTGTAGAAT
*Mi-dbl-1*	ATGGGTATGCTAAACTTGTG
*Mi-dbl-1* R	CGATGCGTCTTCTTCCTCTT
*Mi-sma-6*	CGTGTAATGCTTGGCGTTTT
*Mi-sma-6* R	ATTTCCCCATATCTTCCTTT
*IS-Mi-tph-1*	AGCTACTGGACGTACACGAA
*IS*-*Mi-tph-1* R	TGCATGCCAAGAATTTTTAG
*IS-Mi-dbl-1*	TGGCAGGATTGGATTGTTGC
*IS*-*Mi-dbl-1* R	GGCTTTAACAACGAGCGCAT
*IS-Ce-tph-1*	CCGAACGGAAAACTTGGGGA
*IS-Ce-tph-1* R	CCTGCCAAGAAATCACGAGC
*IS-Ce-dbl-1*	AACAATGATCGATTTCAAATCGAATC
*IS-Ce-dbl-1* R	GGTTCGGACAGGTCACTGAA

### *In vitro* silencing of target genes

Purified PCR products were used as the template to synthesize the dsRNA of each target gene using the MEGAscript™ SP6 Transcription Kit following the method described by Shivakumara et al. (2018) (Thermo Fisher, Waltham, MA, United States). RNA interference (RNAi) treatment of nematodes was performed following a previously described method (Bakhetia et al., 2005) with minor modifications. Briefly, the freshly hatched J2s were immersed in soaking buffer with 1 mg/ml dsRNA supplemented with 0.1 mg/ml FITC, 0.1% resorcinol, 20 mM octopamine, 30 mM spermidine, and 1% gelatin on a rotary mixer in the dark at room temperature for 24 h. Fluorescein isothiocyanate (Beyotime, Shanghai, China) was used as a tracer to assess the uptake efficiency of dsRNA by observing the fluorescence intensity. *Meloidogyne incognita* J2s incubated in the soaking buffer without dsRNA probes were used as the negative control. After that, the soaked nematodes were recovered in the nuclease-free water for another 12 h. Real-time qPCR was used to evaluate the efficiency of the transcriptional knockdown of target genes.

### *In situ* hybridization

Probe preparation and hybridization were performed using the DIG-High Prime DNA Labeling and Detection Starter Kit I (Roche, Basel, Switzerland). The probes of *Mi-tph-1* and *Mi-dbl-1* were generated by PCR using previously designed primers (see Table 1) to amplify a 200–250 base pair region from cDNA cloned in pGEM-TEasy. PCR products were then visualized on a 1.2% (w/v) agarose gel, and sequences were verified as described above. One microgram of purified PCR product was added to an RNAase-free PCR tube and mixed with ddH_2_O to a volume of 16 μl. dsDNA was denatured in a boiling water bath for 10 min. The PCR tube was then quickly inserted into an ice-water mixture for 5 min to prevent the renaturation of DNA. Next, 4 μl of DIG-High Prime was added to the above PCR tube. The probe was then labeled with DIG-11-dUTP after incubation at 37°C for 20 h and at 65°C for 10 min to end the reaction. The labeled probe could be immediately used or stored at −20°C for standby application.

Fixation, permeabilization, probe hybridization, and detection were conducted following the procedures of Kimber et al. (2002) with slight modifications. In short, freshly hatched *M. incognita* J2s were thoroughly washed in sterile water and fixed in 4% paraformaldehyde overnight at 4°C. After fixation, the nematodes were washed twice in M9 buffer and permeabilized using 5 mg/ml proteinase K for 25 min on a rotator at room temperature, followed by incubation in an ice bath for 20 min, incubation in methanol for 1 min (−20°C), and incubation in acetone for 1 min (−20°C).

The permeabilized nematodes were thoroughly washed with M9 buffer and resuspended in 1,000 μl of hybridization buffer for prehybridization at 42°C for 2 h. Five μl of digoxigenin-labeled dsDNA probe was diluted with 50 μl of double distilled water and heat-denatured at 95°C for 10 min, followed by an ice bath for 2 min to maintain the melted state of the probe. The denatured probe was then added to the prehybridization system for hybridization at 42°C overnight, and DIC-labeled DNA was added to the control group. The nematodes were washed two times with 2× saline sodium citrate (SSC) with 0.1% SDS at room temperature and then twice with 0.2 × SSC/0.1% SDS at 42°C, followed by one wash with maleic acid buffer for 3 min at room temperature. Hybridization was conducted for 30 min at room temperature in 1 × blocking solution diluted by maleic acid buffer. The antibody of alkaline phosphatase-conjugated anti-digoxigenin was diluted 1:5,000 by 1 × blocking solution and incubated at room temperature for 2 h, followed by washing with maleic acid buffer for 2 min and 2–5 min by detection buffer. After overnight incubation in a color substrate solution of nitroblue tetrazolium salt and 5-bromo-4-chloro-3-indolyl phosphate (NBT/BCIP) diluted 1:50 by detection buffer, the nematodes were washed twice with 0.1 × M9 buffer for 1 min each wash. The nematode specimens were then mounted on a glass slide covered with a cover slip and observed under a microscope.

### Infection assay of tomato root tips

The infection assay was performed on PF-127 medium to evaluate the effect of the repulsive response induced by different biocontrol bacteria on infection of the plant roots (Dash et al., 2017). Using a pipette, ~300 J2s were added to 1.5 cm posterior to the root tips of tomato seedlings on 23% PF-127 medium in a Petri dish with a diameter of 3 cm. Approximately 5 μl bacterial culture was added near the root tips. Roots were stained by acid fuchsin at 48 h after inoculation, and the number of *M. incognita* J2s that invaded the host plant roots was counted.

### Statistical analysis

At least three technical and biological replicates were performed for all assays. All data were expressed as mean ± standard deviation. Statistical analyses were performed using one-or two-way ANOVA and *t*-tests.

## Results

### Repulsive response of *Meloidogyne incognita* induced by biocontrol bacteria

To determine whether the repulsive response of PPNs could be induced by biocontrol bacteria, a PF-127 chemotactic assay was conducted, and the AI was calculated for *M. incognita* J2s after sensing the volatile molecules secreted by the bacterial strains (Figure 1A). Compared with the negative control (blank media), the lawn-leaving behavior of *M. incognita* was observed after 6 h of exposure to the two biocontrol bacteria, and the AI was 0.40 ± 0.08 against *B. nematocida* B16 and 0.3 ± 0.06 against *B. thuringiensis* Bt79. As the time course was extended, the AI was increased to 0.45 ± 0.09 at 12 h and slightly decreased to 0.38 ± 0.09 at 24 h against *B. nematocida* B16. To *B. thuringiensis* Bt79, the AI was firstly decreased to 0.17 ± 0.03 at 12 h and increased to 0.39 ± 0.12 again at 24 h (Figure 1B). Thus, the PF-127 chemotactic assay demonstrated that *M. incognita* J2s can escape efficiently from the different biocontrol bacteria, which is similar with the defensive response of *C. elegans* against its bacterial pathogens (Zhang et al., 2005).

### Identification of the candidate genes responsible for repulsive response of *Meloidogyne incognita*

The repulsive response of *M. incognita* J2 was observed after 6 h of exposure to biocontol bacteria, suggesting that it might involve learning-associated avoidance. We analyzed the homology of key genes of *C. elegans* in canonical signaling pathways, such as *tph-1* encoding serotonin synthase, *mod-1* encoding the GPCR receptor in the serotonin pathway, and *dbl-1* and *sma-6* encoding the ligand and receptor of the TGF-β pathway, respectively, to the genomic sequence of *M. incognita*. Alignment to these four genes revealed low similarities (30%–67%) in amino acid sequences between *M. incognita* and *C. elegans* (Table 2), which urged us to further validate their roles in repulsive response of *M. incognita*.

**Table 2:** Homology of key genes in canonical pathways related to learning-associated avoidance between *Meloidogyne incognita* and *Caenorhabditis elegans.*

Pathway	Gene name	Homology to *C. elegans*	Gene number in *M. incognita*
Serotonin	*tph-1*	67%	Minc3s00501g13379
	*mod-1*	51%	Minc3s00015g00995
TGF-β	*dbl-1*	30%	Minc3s00127g05444
	*sma-6*	42%	Minc3s01231g21921

### Involvement of candidate genes in the repulsive response of *Meloidogyne incognita*

**Figure 2:**
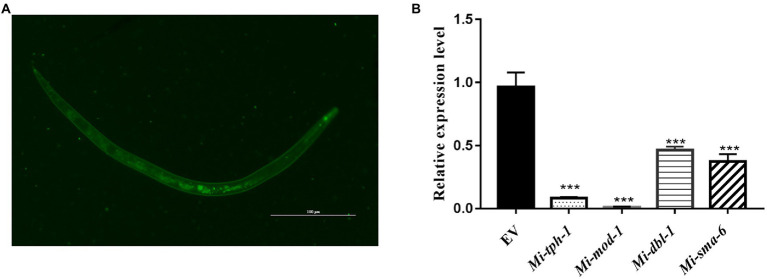
Expression levels of target genes in the RNAi treatment of *Meloidogyne incognita* second-stage juveniles (J2s). **(A)** dsRNA uptake of *M. incognita* J2s determined by the green fluorescence of FITC in the stylet, esophageal gland, and intestine. **(B)** Relative expressional levels of target genes, including *Mi-tph-1*, *Mi-mod-1*, *Mi-dbl-1* and *Mi-sma-6*, quantified by qRT-PCR. EV refers to the control group with empty vector. ^***^p < 0.001.

To validate whether the homologous genes of *M. incognita* play the similar roles in repulsive response against biocontol bacteria, RNAi targeting of *Mi-tph-1* and *Mi-mod-1* in the serotonin pathway, as well as *Mi-dbl-1* and *Mi-sma-6* in the TGF-β pathway, was conducted by soaking *M. incognita* J2s in buffer containing dsRNA probes and FITC. In this reaction system, the green fluorescence of FITC was detected in esophagus and intestinal tract of *M. incognita* J2, suggesting that dsRNA of the target gene was successfully absorbed by *M. incognita* (Figure 2A). To determine the efficiency of gene knockdown, we used qRT-PCR to analyze transcription levels and found that the expression of each gene, including *Mi-tph-1*, *Mi-mod-1, Mi-dbl-1*, and *Mi-sma-6*, was significantly down-regulated as expected (*p* < 0.001; Figure 2B).

**Figure 3:**
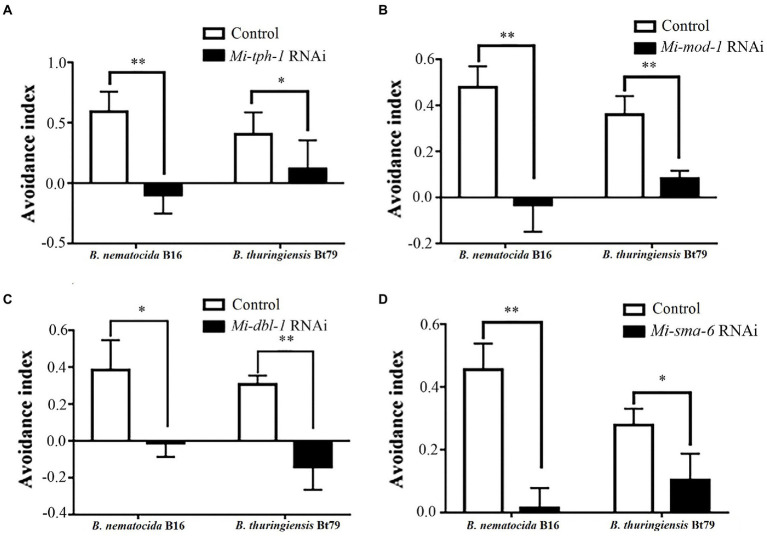
Avoidance index of *Meloidogyne incognita* against different biocontrol bacteria after RNAi treatment of target genes. RNAi of *Mi-tph-1* **(A)**, *Mi-mod-1* **(B)**, *Mi-dbl-1* **(C)**, and *Mi-sma-6* **(D)**. ^*^p < 0.05 and ^**^p < 0.01.

Next, the PF-127 chemotactic assay was performed again for the roles of the candidate genes in repulsive response. Knockdown of genes in either the serotonin or TGF-β pathway weakened the lawn-leaving behavior of *M. incognita* to varying degrees: the AIs of *Mi-tph-1* RNAi decreased from 0.59 ± 0.16 to-0.01 ± 0.15 and from 0.40 ± 0.18 to 0.12 ± 0.24 when *M. incognita* J2s was exposed to *B. nematocida* B16 and *B. thuringiensis* Bt79, respectively (Figure 3A); knockdown of the receptor gene *Mi-mod-1* also attenuated aversive response, and the AIs decreased to-0.03 ± 0.12 and 0.08 ± 0.03 against *B. nematocida* B16 and *B. thuringiensis* Bt79, respectively (Figure 3B). Similarly, RNAi of the other two genes in the TGF-β signaling pathway, *Mi-dbl-1* and *Mi-sma-6*, caused deficiencies in the lawn-leaving behavior against *B. nematocida* and *B. thuringiensis* (Figures 3C,D). Overall, our data indicate that both the serotonin and TGF-β pathways of *M. incognita* play the same roles in the repulsive response to biocontrol bacteria as in *C. elegans* despite their relatively low homology of gene sequences.

**Figure 4:**
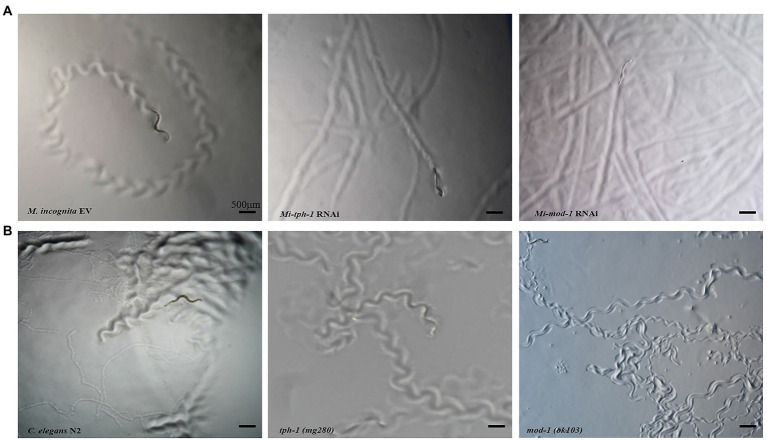
Effects of inactivating the serotonin signaling pathway on the movement trajectory of *Meloidogyne incognita*. **(A)** Movement traces of the control with empty vector, *Mi-tph-1* RNAi, and *Mi-mod-1* RNAi of *M. incognita*. **(B)** Movement traces of the wild type nematodes (N2), *tph-1*(*mg280*), and *mod-1*(*ok103*) of *Caenorhabditis elegans*.

RNAi of *Mi-tph-1* and *Mi-mod-1* also altered the movements of *M. incognita*, and no similar effect has been described for alterations to the serotonin pathway of *C. elegans*. To confirm the potentially new function of the serotonin pathway in *M. incognita*, we tracked the movements of nematodes with *Mi-tph-1* and *Mi-mod-1* knocked down by RNAi. Compared with the control group of *M. incognita* with only empty vector, which displayed crooked movement trajectories on PF-127 medium, the movement patterns of *M. incognita* with either *Mi-tph-1* or *Mi-mod-1* knocked down were altered; specifically, the swing amplitude of the body was reduced, and the traces of the nematodes were more flattened (Figure 4A). No differences in body rigidity and movement patterns were observed in N2, *tph-1(mg280)*, and *mod-1(ok103)* of *C. elegans* (Figure 4B).

### Neuronal localization of the serotonin and TGF-β pathways in *Meloidogyne incognita*

**Figure 5:**
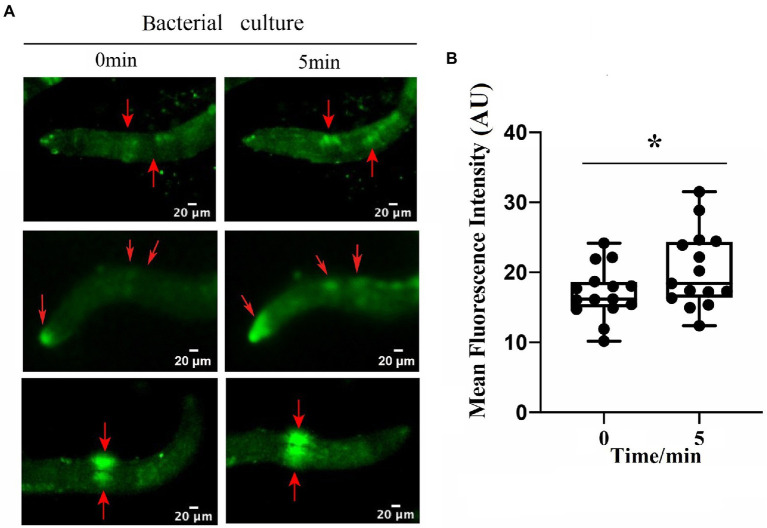
Involvement of multiple amphid neurons in the repulsive response of *Meloidogyne incognita*. **(A)** Images of intracellular Ca^2+^ measurements on the amphid neurons using Fura-2-AM. **(B)** Quantitative analysis to the mean fluorescence intensity in the amphid neurons before and after adding the bacterial culture (*n* = 15). ^*^p < 0.05.

To identify the neuron(s) involved in the repulsive response of *M. incognita* against biocontrol bacteria, we measured intracellular changes in Ca^2+^ using Fura-2-AM imaging. Once the bacterial culture of either *B. nematocidal* B16 or *B. thuringiensis* Bt79 was sensed by *M. incognita* J2 and the repulsive response was stimulated, the fluorescence enhancement of Ca^2+^ was detected in multiple amphid neurons of *M. incognita* J2 (Figure 5A), which was further supported by a statistical significance of the average fluorescence intensities within the amphid neurons at 0 min and 5 min (Figure 5B).

**Figure 6:**
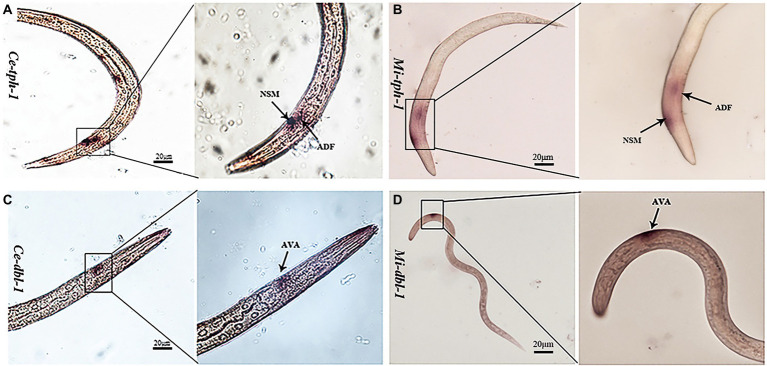
Comparisons of the neuronal localization. *In situ* hybridization with the DIG-labeled probes of *Ce-tph-1 in Caenorhabditis elegans* **(A)** and *Mi-tph-1* in *Meloidogyne incognita* **(B)** on NSM and ADF neurons, *Ce-dbl-1 in C. elegans* **(C)** and *Mi-dbl-1* in *M. incognita* **(D)** on AVA neurons.

As previous studies have reported that the aversive behavior of *C. elegans* depends on the functioning of *tph-1* in ADF neurons and *dbl-1* in AVA neurons (Zhang et al., 2005; Zhang and Zhang, 2012), we characterized their neuronal localization in *M. incognita*. ISH revealed that these two genes exhibited similar expressional patterns in the amphid neurons, with *Mi-tph-1* mainly localized in ADF/NSM neurons (Figures 6A,B) and *Mi-dbl-1* in AVA neurons (Figures 6C,D).

### Repulsive response of *Meloidogyne incognita* suppresses their invasion into host plants

**Figure 7:**
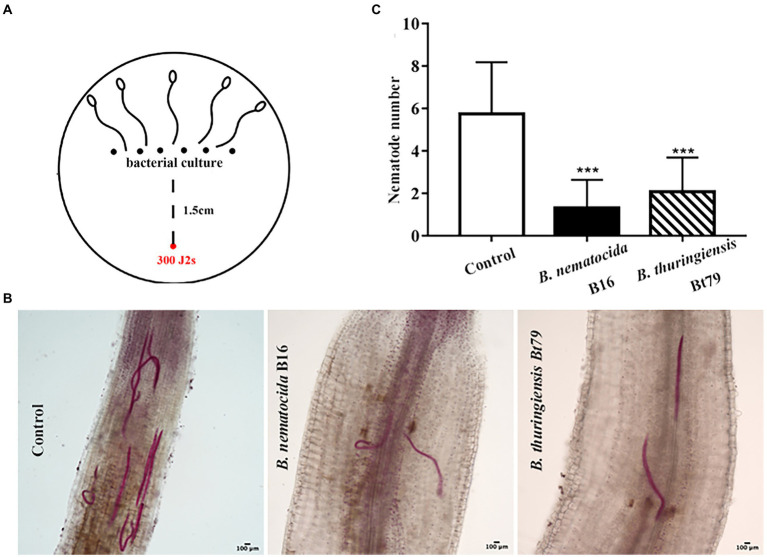
Induction of the repulsive response of *Meloidogyne incognita* by biocontrol bacteria suppresses the invasion of *M. incognita* into host plants. **(A)** Schematic diagram of the assay for evaluating the effects of repulsive response on the invasion of *M. incognita* second-stage juveniles (J2s) into tomato roots. **(B)** Stained nematodes that have invaded tomato roots after 48 h. **(C)** The numbers of nematodes inside tomato roots (*n* = 10). ^***^p < 0.001.

Infection experiments was designed to investigate whether the repulsive response affects the colonization of PPNs within host plants or alters the efficiency of biocontrol bacteria (Figure 7A). Treatment of either *B. nematocida* B16 or *B. thuringiensis* Bt79 significantly decreased the number of nematodes inside tomato roots 48 h (Figure 7B). Statistical analysis to 10 samples in each treatment suggested that the average numbers of nematodes in the root tips were 1.30 ± 1.34 and 2.07 ± 1.62 in the groups treated with *B. nematocida* B16 and *B. thuringiensis* Bt79, which were 4.42 and 2.78 times lower than that of the control (5.75 ± 2.44), respectively (Figure 7C).

## Discussion

In natural ecosystems, rhizosphere microbiota participates in interactions between nematodes and plants. The symbiotic bacteria of plants, such as *Bacterillus* and *Pseudomonas*, synthesize hundreds of compounds to either stimulate plant growth or enhance the resistance to nematodes (Santoyo, 2021). Additionally, a few non-virulence bacteria, acting as the food of bacterivorous nematodes, have developed a strategy to mobilize their ally of nematode-trapping fungi to help them kill nematodes (Wang et al., 2014).

The biocontrol bacteria, *B. nematocidal* and *B. thuringiensis*, can induce the repulsive response of *M. incognita* J2s after 6 h of exposure to inhibit their invasion of host plants. But what’s interesting is that the AI index for *B. thuringiensis* Bt79 was once decreased at 12 h, and then continued to increase at 24 h. This change trend seems different from that of *B. nematocidal* B16, which retains a constant increase at all time points. It may be due to the differential volatile compounds between *B. nematocidal* and *B. thuringiensis*.

The pathogenic bacterium *P. aeruginosa* PA14 can stimulate learning-associated avoidance in *C. elegans* after 4 h of exposure, and this might increase their survival rate during infection (Zhang et al., 2005). A few molecular mechanisms have been suggested to underlie these behaviors. First, serotonin from ADF chemosensory neurons is increased by transcriptional and post-transcriptional mechanisms. After binding to its receptor MOD-1, a serotonin-gated chloride channel localized to the sensory interneurons AIY and AIZ, serotonin promotes the aversive learning of *C. elegans* against *P. aeruginosa* PA14 (Zhang et al., 2005; Melo and Ruvkun, 2012). The module DBL-1/SMA-6 in the TGF-β signaling pathway is also thought to be required for the lawn-escaping behavior of *C. elegans*. After aversive training with *P. aeruginosa* PA14, the repressed activity of AVA interneurons triggers an increase in the expression of the ligand DBL-1, which binds to the type I TGF-β receptor SMA-6 on ASI neurons, promotes olfactory plasticity, and induces a strong aversive response (Zhang and Zhang, 2012). In light of differences in the genomic sequences between *M. incognita* and *C. elegans* and limitations in current research techniques, the genes, molecular pathways, and neural circuits involved in the repulsive response of *M. incognita* require further study.

Sequence alignment of the genes involved in the above two classical pathways between *C. elegans* and *M. incognita* revealed a similarity >50% for genes in the serotonin pathway and 30%–42% for genes in the TGF-β signaling pathway. Silencing of the key genes in both pathways decreased the AIs of *M. incognita* J2 against biocontrol bacteria, which confirmed that they played similar roles in the repulsive response of *M. incognita*. Ca^2+^ imaging with Fura-2-AM and ISH showed that both sets of genes functioned in the same neural circuits in *M. incognita* as in *C. elegans*. However, the contributions of these signaling pathways vary to the repulsive response induced by the different biocontrol bacteria. Additionally, a novel role of serotonin pathway has also been suggested to modulate the muscle tone of *M. incognita*, as decreased body swing amplitude and more flattened traces were observed following RNAi of *Mi-tph-1* and *Mi-mod-1*, and such changes are absent in *C. elegans*. We speculate that this might be explained by the fact that the reduced genome of *M. incognita* possesses genes that are responsible for more biological activities.

The repulsive response of *M. incognita* induced by biocontrol bacteria likely affects interspecific interactions among bacteria, PPNs, and host plants. In agriculture, the control of PPNs is fairly difficult because the nematodes generally inhabit soil and attack the underground parts of plants. Chemical nematicides have long been thought to be the most effective approach for controlling PPNs. Due to their high toxicity, as well as the ease with which they form residues and can be abused, the use of chemical nematicides can have deleterious effects on the environment and cause the serious pollution for agricultural products. Therefore, biological control, which is a more environmentally friendly alternative that exploits the interactions between nematode-antagonistic microorganisms and their hosts, provides a more robust complementary approach. Nematode-antagonistic microorganisms employ different strategies to alleviate PPN infections in plants. Theoretical studies of their interactions are of great value to improve the efficacy of biocontrol by enhancing the virulence factors of biocontrol microorganisms and weakening the defensive response of PPNs.

Since semiochemicals have been successfully used to control pests (Park et al., 2003; Lee et al., 2004), and the infection efficiencies of *M. incognita* can be greatly reduced *via* induction of the repulsive response by biocontrol bacteria, we believe the results of our study provide new insights into the biocontrol control of PPNs.

## Data availability statement

The original contributions presented in the study are included in the article/supplementary material, further inquiries can be directed to the corresponding author.

## Author contributions

XH conceived and designed the study. YZ and QZ conducted experiments, searched the literature and wrote the manuscript. XH, CZ, and KZ revised the manuscript. XH and CZ supplied funding. All authors contributed to the article and approved the submitted version.

## Funding

This work was supported by the National Natural Science Foundation of China (grant number 32170184, 32060632, and U1802233) and the Department of Science and Technology of Yunnan Province (grant number 2019FA046).

## Conflict of interest

The authors declare that the research was conducted in the absence of any commercial or financial relationships that could be construed as a potential conflict of interest.

## Publisher’s note

All claims expressed in this article are solely those of the authors and do not necessarily represent those of their affiliated organizations, or those of the publisher, the editors and the reviewers. Any product that may be evaluated in this article, or claim that may be made by its manufacturer, is not guaranteed or endorsed by the publisher.
